# Programmable somatosensory soft robots

**DOI:** 10.1038/s41528-026-00558-0

**Published:** 2026-03-07

**Authors:** Antonia Georgopoulou, Malena Aguiriano Calvo, Lorenzo Lucherini, Sudong Lee, Josie Hughes, Esther Amstad

**Affiliations:** 1 Soft Materials Laboratory, Institute of Materials (SMaL), École Polytechnique Fédérale de Lausanne, Lausanne, Switzerland; 2 Swiss National Center for Competence in Research (NCCR) Bio-inspired Materials, University of Fribourg, Fribourg, Switzerland; 3 CREATE Lab, Institute of Mechanical Engineering, École Polytechnique Fédérale de Lausanne, Lausanne, Switzerland

**Keywords:** Engineering, Materials science

## Abstract

Robotic intelligence has advanced greatly in the past decade. Nevertheless, integrating embodied intelligent and responsive behavior into soft robotic systems remains challenging because it typically requires bulky hardware for environmental feedback and decision-making. While soft materials like poly(N-isopropylacrylamide) (PNIPAM) offer potential for simplified material-based actuation through temperature-responsive motion, their slow response and high energy demands limit their use in closed-loop control systems. To overcome this limitation, we present soft PNIPAM-based actuators with integrated hydrogel-based Joule heating, enabling localized actuation without significantly altering the temperature within 1 cm of the actuator. The potential of the material is demonstrated by processing it into a soft gripper that can lift up to three-fold its own weight with integrated capability to adjust its actuation in response to the gripped object. This design is well-suited for energy-efficient manipulation and sorting of delicate items, such as those found in automated packaging systems.

## Introduction

Significant advances in intelligent behaviors of soft robotics were achieved by embedded sensing and model-free control systems. Unfortunately, the physical capacity of soft robots to sense and actuate remains limited^1–3^. The ability to collect and process information from the robot’s environment for feedback and decision-making often depends on complex algorithms and hardware components^4–7^. Responsive materials offer a promising alternative to actuate robotic systems without the need for complicated hardware and control methods by changing environmental conditions^8–11^. A prominent example is the thermo-responsive hydrogel poly(N-isopropylacrylamide) (PNIPAM), which possesses a lower critical solution temperature (LCST) around 32 °C^12,13^. The reversible swelling behavior of PNIPAM can be harnessed to induce repeated motion, like bending^14–19^.

Establishing closed-loop control in soft robotic systems requires a direct and electronically accessible link between the actuator’s body and its control architecture^4,20,21^. For PNIPAM-based actuators, this link has been difficult to achieve because traditional PNIPAM actuation relies on being exposed to hot environments, such as hot fluids. This mode of activation prohibits the use of these materials in large-scale environments whose temperature cannot be readily changed, results in long response times, and imposes substantial energy costs^22^. These constraints have limited the use of PNIPAM in soft robotics^23,24^. Alternative approaches, such as heat-radiation-driven actuation, are slow and lack real-time electrical control of deformation^25,26^. Consequently, PNIPAM hydrogel actuators with integrated, electronically controlled, closed-loop sensing and actuation capabilities have not yet been demonstrated.

Electrical energy can be converted into heat through resistive losses, called Joule heating^11,27,28^. Joule heating elements can be integrated in closed-loop control setups in line with resistive sensors. Traditional Joule heaters are composed of rigid metals or alloys, which are suboptimal for soft actuators because they stiffen them. Flexible Joule heating elements have been fabricated from polymers and conductive fillers. However, these elements are often composed of rigid polymers or thermoplastic elastomers with a Shore hardness of 85 A, which stiffen the overall structures^29–32^. Hydrogel-based flexible Joule heaters used as anti-freeze devices that restore the temperature of e-skins from subzero to room temperature have been introduced. However, they have never been employed to regulate thermally responsive actuators^33^.

**Fig. 1:**
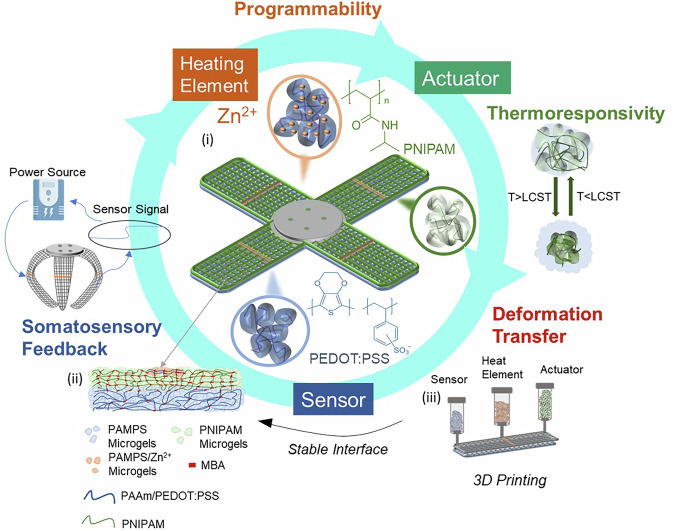
Soft robotic gripper exhibiting a closed loop between sensing and actuation. (**i**) The gripper is composed of two types of double network granular hydrogels (DNGHs): The actuator is made of Poly-N-isopropylacrylamide (PNIPAM) microgels connected through a PNIPAM secondary network. The sensor is composed of poly(2-acrylamido-2-methyl-1-propanesulfonic acid) (PAMPS) microgels connected through a polyacrylamide (PAAm) secondary network blended with poly(3,4-ethylenedioxythiophene)-poly(styrenesulfonate) PEDOT:PSS. To convert certain locations of the sensor into a Joule heater, they are functionalized with PEDOT:PSS and Zn^2+^ to impart them electronic and ionic conductivity. (**ii**) The sensor and actuator are covalently connected, resulting in firm material interfaces. (**iii**) Leveraging the granular structure of the material, it can be direct ink written into intricate structures with locally varying compositions.

In this study, we introduce soft, thermo-responsive hydrogel-based actuators with incorporated Joule heating elements, which enable an energy-efficient closed-loop control of the actuation. We demonstrate the potential of these materials by 3D printing them into a fully integrated soft robotic gripper, capable of size-selectively lifting desired fruits. The gripper incorporates PNIPAM actuators, piezoresistive sensors, and Joule heaters composed of electronically and ionically conductive hydrogels. The localized heat, generated if currents flow through these conductive tracks, raises the temperature locally above the LCST of PNIPAM, thereby initiating its actuation within 35 s. Importantly, the Shore hardness of the Joule heater is similar to that of PNIPAM, preventing any hardness mismatch within our actuator that could negatively impact its lifespan. The embedded sensor converts the actuator’s deformation into proprioceptive and contact information, which is used to control the heating element for closed-loop actuation, as shown schematically in Fig. 1. These features enable the soft robot to sense its own deformation and detect object contact, which are important ingredients towards embedding somatosensory capability into soft robots. We foresee the combination of electronic programmability, somatosensory feedback, and the flexibility in the processing of these materials provided by their 3D printability to enable the fabrication of closed-loop soft robots that are more flexible, energy efficient, and can be used in temperature-sensitive environments, including biological milieus.

## Results

### Influence of actuator stiffness on the bending angle

**Fig. 2:**
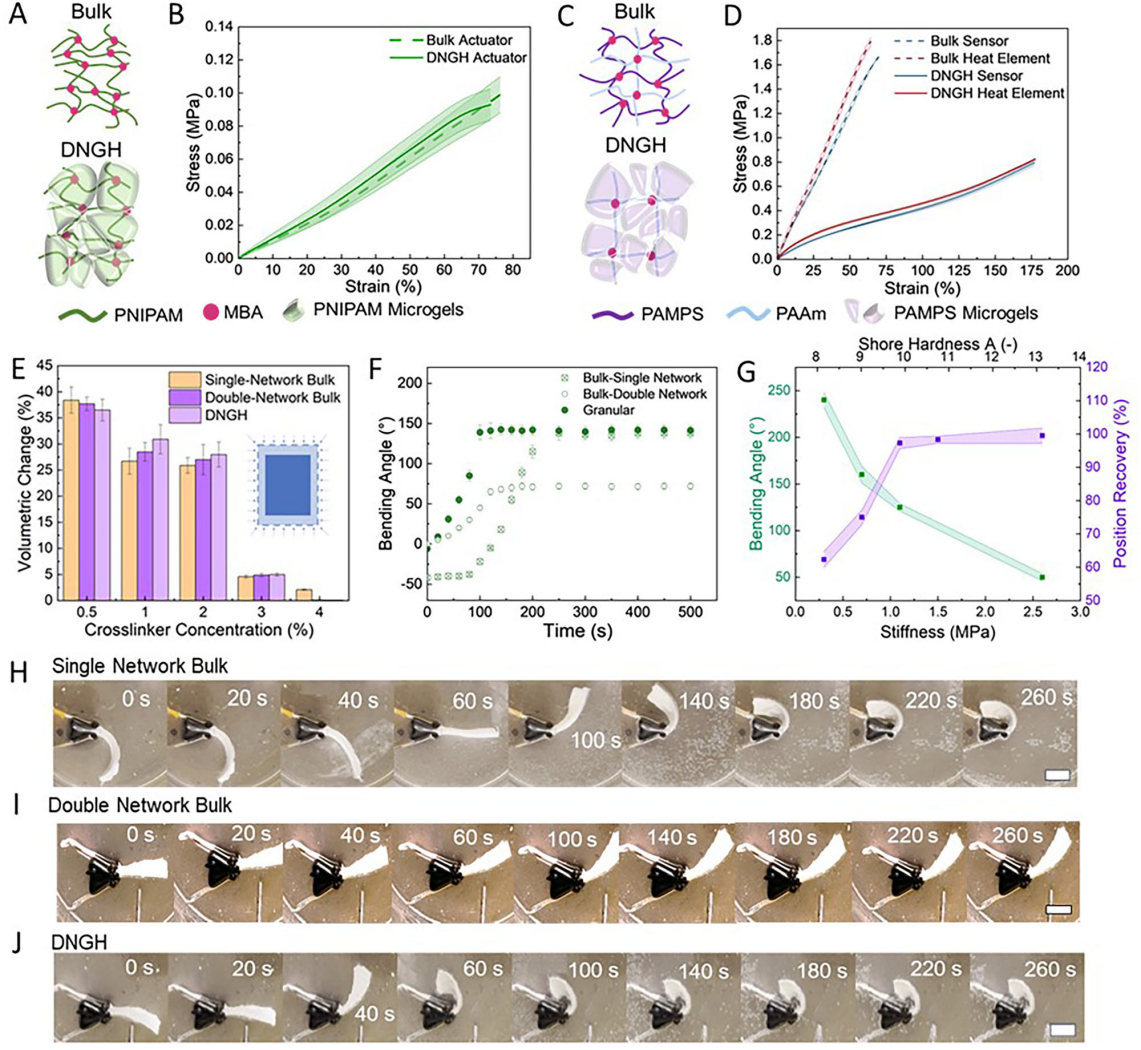
Actuation of DNGHs and bulk counterparts. Schematic illustration of the double network bulk **A** actuator and **C** sensor with their DNGH counterpart (**B**–**D**). The corresponding stress-strain curves of double network bulk (dotted line) and DNGH (straight line) based **B** actuators, **D** sensors (blue), and heating elements (red). **E** Volumetric change of single-network bulk (orange), double network bulk (purple), and DNGH (violet) actuators as a function of the crosslinker concentration within PNIPAM. The crosslinker concentration within microgels and the 2^nd^ network is identical. **F** Actuation kinetics of single-network, double-network, and DNGH bilayers. **G** Maximum bending angle of DNGH bilayers at 55 °C as a function of the crosslinking density within PNIPAM (green) and recovery of their initial position (purple) at 25 °C. Timelapse of bilayers made of: **H** single-network PNIPAM bulk (scale bar 1 cm), **J** double network PNIPAM bulk (scale bar 1 cm), and **I** DNGH PNIPAM (scale bar 1 cm) if heated to 55 °C.

To create a somatosensory thermally responsive actuator, we fabricate PNIPAM microgels by fragmenting single-network PNIPAM hydrogels. The fragments, with an average diagonal of 11 μm, are soaked in an aqueous solution containing NIPAM. The precursor-loaded PNIPAM microgels are jammed before they are cast or 3D printed into the desired shape. The structures are rigidified through polymerization of the NIPAM precursors to form a 2^nd^ percolating hydrogel that interpenetrates the microgels and covalently connects them, resulting in thermally responsive DNGHs, which we use as an actuator (DNGH-A), as shown in Fig. 2A.

To assess the influence of the microstructure of the DNGH-A on its mechanical properties, we perform tensile tests on DNGHs and bulk double-network gels of the same composition but lacking any microstructure, as schematically shown in Fig. S1A. Bulk double network PNIPAM and DNGH-A exhibit an ultimate strength of 0.1 MPa, elongation at break of 75%, and a Young’s modulus of 0.12 MPa, as shown in Fig. 2B. Because the 2^nd^ network has the same crosslinker concentration as the 1^st^ counterpart, the microstructure of DNGH-A does not significantly affect the stiffness or work of fracture of the actuator. We select identical crosslinker concentrations for both phases because higher crosslinker concentrations in the microfragments result in the loss of thermoresponsivity in the DNGOGs and a reduced strain at break, as shown in Fig. S2. Lower crosslinker concentrations in the 2^nd^ network compromises the integrity of DNGOGs.

To fabricate piezoresistive sensors, we impart the DNGHs electrical conductivity by incubating PAMPS microgels in an aqueous solution containing AAm and PEDOT:PSS, as shown in Fig. 2C. We chose PAMPS microgels because they have a higher degree of swelling in water compared to PNIPAM, such that they take up a larger volume of precursors, translating into superior work of fracture and strain at break of the resulting DNGHs, as shown in Fig. S3. Precursor-loaded PNIPAM fragments are jammed and processed into the desired shape. The shape is rigidified by polymerizing the AAm to form a 2^nd^ hydrogel network, resulting in a piezoresistive DNGH Sensor (DNGH-S).

To endow electronic programmability to our DNGH-A, we increase the conductivity of the DNGH-S to use it as a flexible Joule Heating element (DNGH-H). To achieve this goal, we add zinc chloride (ZnCl_2_) to the precursor solution of the 2^nd^ network. To test the influence of the microstructure of the DNGH-H on its mechanical properties, we perform tensile tests on it and compare the results to those of bulk samples possessing the identical overall composition. The Young’s modulus of ZnCl_2_-free DNGH-A is 0.7 MPa. The addition of ZnCl_2_, which converts DNGH-As into DNGH-Hs, increases the Young’s modulus to 1 MPa. The ultimate strength and strain at break do not significantly change upon ZnCl_2_ addition, remaining at 0.78 MPa and 170%, respectively, as shown in Fig. 2D. These results indicate that the DNGH-H maintains softness and extensibility, despite the added ionic conductivity. Because the 2^nd^ network is relatively soft, the Young’s modulus of bulk hydrogels is much higher than that of DNGHs, 2.7 MPa. These results indicate that the microstructure of the heating element significantly softens it, such that its stiffness is within the same order of magnitude as that of DNGH-As.

To transform the volumetric changes of PNIPAM into a bending motion required for gripping, we formulate bilayer structures by combining an active layer made of DNGH-A with a passive layer made of DNGH-S, as shown in Fig. S1B. The DNGH-S and DNGH-A are simultaneously solidified through the free radical polymerization of acrylamide-based precursors to form a 2^nd^ network. Hence, we expect the two types of 2^nd^ networks to covalently connect, thereby forming strong material interfaces.

To maximize the bending angle, a large volumetric change of the DNGH-A is required. We expect the volumetric change to depend on the stiffness of the DNGH-A. To evaluate the effect of the stiffness of the DNGH-A on its actuation, we vary the crosslinker concentration within the PNIPAM microgels of the DNGH-A. When we increase the crosslinker concentration from 1 to 2 wt%, the Young’s modulus of the DNGH-A increases from 0.09 to 0.16 MPa, as shown in Fig. S4. This increase in stiffness minimally reduces the temperature-induced volumetric change from 28 to 25%, as shown in Fig. 2E. Similar results are obtained for bulk actuators of the same composition, as shown in Fig. 2E, suggesting that the microstructure does not influence the thermo-response of the DNGH-A. Any further increase of the crosslinker concentration reduces the temperature-induced volumetric change of the hydrogel, rendering it unsuitable for actuation. Therefore, we fix the crosslinker concentration to 2 wt% for the remainder of this study.

To assess the influence of the temperature-induced volumetric change of the actuator on the bending of the bilayer, we fabricate a bilayer composed of a 1.5 mm thick DNGH-A and a 1.5 mm thick passive DNGH-S. This bilayer bends 150°. The same bending angle can be achieved by single-network PNIPAM actuators of the same thickness. This is in stark contrast to a bilayer composed of double network bulk PNIPAM, that only bends to 52°, as shown in Fig. 2F. We assign the much lower bending angle of bulk double network hydrogels to the increased stiffness of the passive layer, since bulk hydrogels are significantly stiffer than their DNGH counterparts, as shown in Fig. 2G. Remarkably, DNGH-based actuators maintain a horizontal position at rest, in stark contrast to bilayers based on single network bulk hydrogels despite their similar stiffnesses, as shown in Fig. 2G. This comparison highlights an important advantage of DNGHs: They can maintain a horizontal position at rest while reaching a high bending angle if actuated, a combination that cannot be achieved with bulk samples of similar compositions.

The performance of many actuators depends on their response time. Single network bulk bilayers reach the maximum bending angle after 215 s. DNGH-based bilayers reach the maximum bending angle much faster: within 95 s, as shown in Fig. 2F. We assign the faster response of DNGH-A to their initially straight form, as shown in Fig. 2I, J, in stark contrast to the single network bulk bilayers, which initially attain a negative deflection, as shown in Fig. 2H. This negative deflection must be compensated before the actual actuation takes place, prolonging the actuation time.

To assess the influence of the stiffness of the passive layer on the bending angle of the bilayer, we increase the stiffness of the DNGH-S by increasing the crosslinker concentration in the PAAm network, while the stiffness of the DNGH-A is kept constant. We do not vary the stiffness of the microgels in the DNGH-S, because softer particles lead to undesirable signal drift in the sensor response due to large stress-strain hysteresis in DNGH-S^34^. As expected, the maximum bending angle decreases from 250° to 125° if the stiffness of the DNGH-S increases from 0.5 to 1.2 MPa, translating into an increase in Shore hardness from 8 to 10 A, as shown in Fig. 2G. A further increase in stiffness to 2.5 MPa decreases the bending angle to 51°. This result suggests that a soft passive layer is better suited for bending applications. However, too soft passive layers prevent the recovery of the initial shape if cooled to room temperature. Indeed, bilayers containing a passive DNGH-S layer with a stiffness of 0.47 MPa only recover 70% of the horizontal position, as shown in Fig. S4 and Movie 1. By contrast, bilayers containing DNGH-Ss with a stiffness of 1.2 MPa or higher return to their original shape if cooled to 25 °C. Based on these results, we fix the stiffness of the passive DNGH-S to 1.2 MPa.

### Flexible joule heating element

**Fig. 3:**
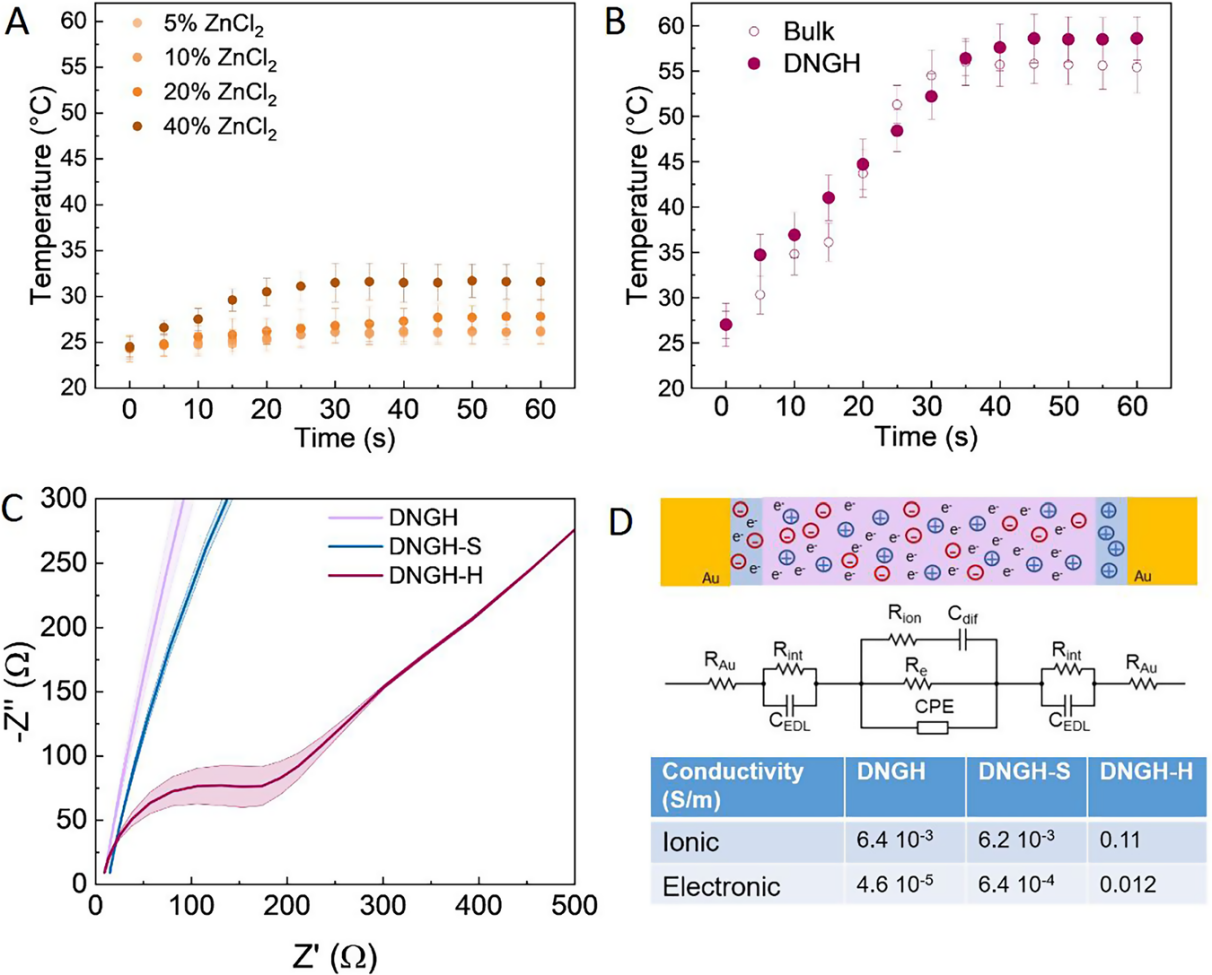
Characterization of the Joule heating element, DNGH-H. **A** Temperature evolution of PAAm gels functionalized with 5 wt% (very light), 10 wt% (light), 20 wt% (medium), 40 wt% (dark) ZnCl_2_, if connected to a 5 V source. **B** Temperature of double network PAMPS/PAAm bulk gels (white) and PAMPS/PAAm DNGH-H (magenta) functionalized with 40 wt% ZnCl_2_ and 0.027 wt% PEDOT:PSS measured at the surface of the gels. **C** Nyquist plots for DNGH-H containing 40 wt% ZnCl_2_ and 0.027 wt% PEDOT:PSS and DNGH-S containing 0.027 wt% PEDOT:PSS. **D** Schematic and equivalent electric circuit of the DNGH, electrodes (R_Au_) and interface (R_int_ and C_EDL_) measured with impedance spectroscopy. In the equivalent circuit models, R_e_ represents electronic resistance, R_ion_ ionic resistance, C_dif_ the diffusion limited properties of the ionic conduction, constant phase element (CPE) accounts for geometric inhomogeneous or imperfect capacitance of the system. The corresponding Bode plots are shown in Fig. S8.

To minimize the amount of energy needed for actuation and enhance the applicability of our DNGH-A in environments that cannot be easily heated, we generate heat near the thermo-responsive PNIPAM-based DNGH-A. According to Joule’s first law, the heat generation in resistive heaters is influenced by their electrical conductivity. To investigate how conductivity affects the heat generation of our DNGH-H, we vary the ion concentration in the acrylamide precursor solution and measure the temperature of the resulting DNGH-H when 5 V is applied. The surface temperature of 2 cm thick DNGH-H gels increases during the first 30 s, whereafter it plateaus independent of the concentration of ZnCl_2_ in the precursor solution, as shown in Fig. 3A. However, the plateau temperature increases with increasing ZnCl_2_ concentration: An increase in the ZnCl₂ concentration from 20 to 40 wt% raises the plateau temperature from 25 to 30.5 °C. Under our fixed-voltage operating conditions, this trend is consistent with Joule’s law, as higher ZnCl₂ concentrations decrease the resistance of the hydrogel heater, thereby increasing the dissipated power^29,30^. Unfortunately, even the highest plateau temperature we obtain, 30.5 °C, is below the lower critical solution temperature (LCST) of PNIPAM, such that it cannot induce actuation of the DNGH-A.

To enhance the Joule heating effect, we functionalize the DNGH-H with an electrically conductive polymer poly(3,4-ethylenedioxythiophene)-poly(styrenesulfonate) (PEDOT:PSS). The thermal conductivity of PEDOT:PSS is low, preventing its use as a Joule heating element, as shown in Fig. S5. However, the addition of PEDOT:PSS to the ZnCl_2_ functionalized DNGH-A increases the plateau temperature to 52 °C if connected to a 5 V source, as shown in Fig. 3B. This temperature is well above the LCST of PNIPAM, such that the actuator readily shrinks if 5 V is applied, as shown in Fig. S6. We obtain a similar temperature evolution for bulk hydrogels possessing the identical composition but lacking any microstructure, as shown in Fig. 3B. These results indicate that the microstructure does not significantly affect the Joule heating efficiency.

To verify that ionic and electronic conductivity are present in our system, we perform electrical impedance measurements on the DNGH-H^35^. The low PEDOT:PSS concentration present in DNGH-Hs is sufficient to impart them electrical conductivity because the polymer accumulates in their interstitial spaces^34^. To differentiate between effects caused by PEDOT:PSS and ZnCl_2_, we perform the same test on the sensor DNGH-S that contains 0.027 wt% PEDOT:PSS but lacks ZnCl_2_, and DNGH, lacking any functionalization. DNGHs and DNGH-Ss are resistors, as indicated by the straight line in the Nyquist plots in Fig. 3C. By contrast, DNGH-Hs exhibit a semicircle at low frequencies, indicative of a resistive behavior with an additional time constant, which is associated with ionic conductivity^35^. We observe a similar profile of the Nyquist plots in double network bulk materials, even though the values of the electrical impedance are two orders of magnitude higher than those of their granular counterparts, as shown in Fig. S9. This difference is possibly due to the reduced polymer density within the interstitial spaces of DNGH-Hs, which increases the diffusivity of ions within these locations and hence, their conductivity.

To calculate the electronic and ionic conductivity of our DNGH, we model the Nyquist plots with the equivalent electronic circuit indicated in Fig. 3D. The circuit contains elements that simulate the electronic, ionic conductivity of the DNGH-H, the resistivity and capacity of the gold electrodes, and interfaces between the sample and gold electrodes^36^. The addition of 40 wt% Zn^2+^ increases the ionic conductivity by two orders of magnitude from 6.2 ∙ 10^−3 ^S/m to 4.4 ∙ 10^−1 ^S/m, as shown in Fig. 3D. Interestingly, the addition of 40 wt% Zn^2+^ also increases the electrical conductivity from 6.4 ∙ 10^−4 ^S/m to 1.2 ∙ 10^−2 ^S/m. The increase in electrical conductivity might be caused by the Zn^2+^ induced dissociation of PEDOT from PSS. This dissociation leads to the precipitation of PEDOT that increases its concentration within the interstitial spaces of DNGHs, thereby facilitating the formation of a percolating network and leading to a higher electrical conductivity. To test this hypothesis, we perform UV-VIS spectroscopy on the supernatant of jammed microgels. Indeed, the supernatant of jammed microgels encompassing PEDOT:PSS and Zn^2+^ ions exhibits an absorption peak at 240 nm, indicative of PSS^37^, and one at 349 nm, indicative of Zn^2+^ ions^38^, as shown in Fig. S10a. By contrast, the supernatant of jammed microgels encompassing PEDOT:PSS exhibits the peak at 224 nm, indicative of PEDOT, and 240 nm, indicative of PSS, as shown in Fig. S10a. The hypothesis that Zn^2+^ ions dissociate PEDOT:PSS complexes is further confirmed by the visual inspection of the supernatant of DNGH-S, which exhibits a blue color indicative of a high PEDOT concentration. In contrast, the supernatant of DNGH-H is transparent, indicating that the vast majority of PEDOT has been incorporated in the DNGH-H, as shown in Fig. S10b.

### Joule heating-induced actuation

**Fig. 4:**
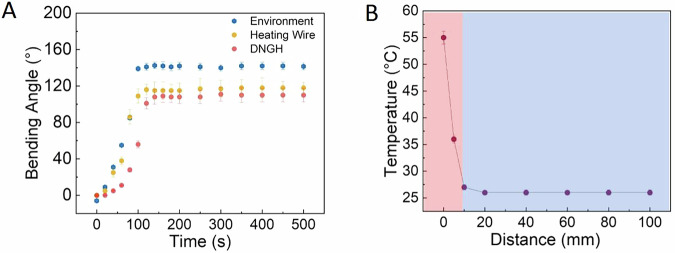
Influence of the heating element on actuation. **A** DNGH-As activated by immersing them into 55 °C water (blue), through an integrated metallic heating wire (orange), or DNGH-H (red) at a solution temperature of 25 °C. **B** Variation of temperature with the distance from the surface of the DNGH-A with integrated DNGH-H immersed in 25 °C water measured with an infrared camera.

To evaluate the capability of the DNGH-H Joule heating element to induce bending of the composite actuator, we assess the evolution of the bending angle of bilayer structures consisting of DNGH-A and DNGH-S. We immerse the bilayer in 500 mL of aqueous solution at 25 °C and apply 5 V. The bilayer, with a total thickness of 3 cm, reaches a maximum bending angle of 140° within 95 s when immersed in 55 °C water. When using a commercial metallic heating wire, the maximum bending angle decreases to 109°. More limiting is the structural damage of the PNIPAM-based hydrogel, caused by the stiff metal wire during bending. This localized damage can be avoided by exchanging the rather stiff metal wire with the flexible DNGH-H. Notably, with a single flexible DNGH-H embedded as a Joule heater, the actuator achieves a similar bending angle of 105°, as shown in Fig. 4A. Note that the maximum bending angle is smaller, 105°, if the actuation is caused by Joule heating. Yet, the heating elements enable programmed closed-loop control of the actuation. An additional benefit of the localized heating is that the temperature of the surrounding environment remains constant. This feature renders the actuator suitable for biomedical applications without risking temperature-induced damage to tissue, in contrast to PNIPAM-based actuators that rely on bulk heating. Indeed, the temperature of the water measured 10 mm apart from the active actuator reaches only 27 °C, as shown in Fig. 4B. The localized heating significantly reduces the power consumption of the actuator.

### Rheological characterization and 3D printing

**Fig. 5:**
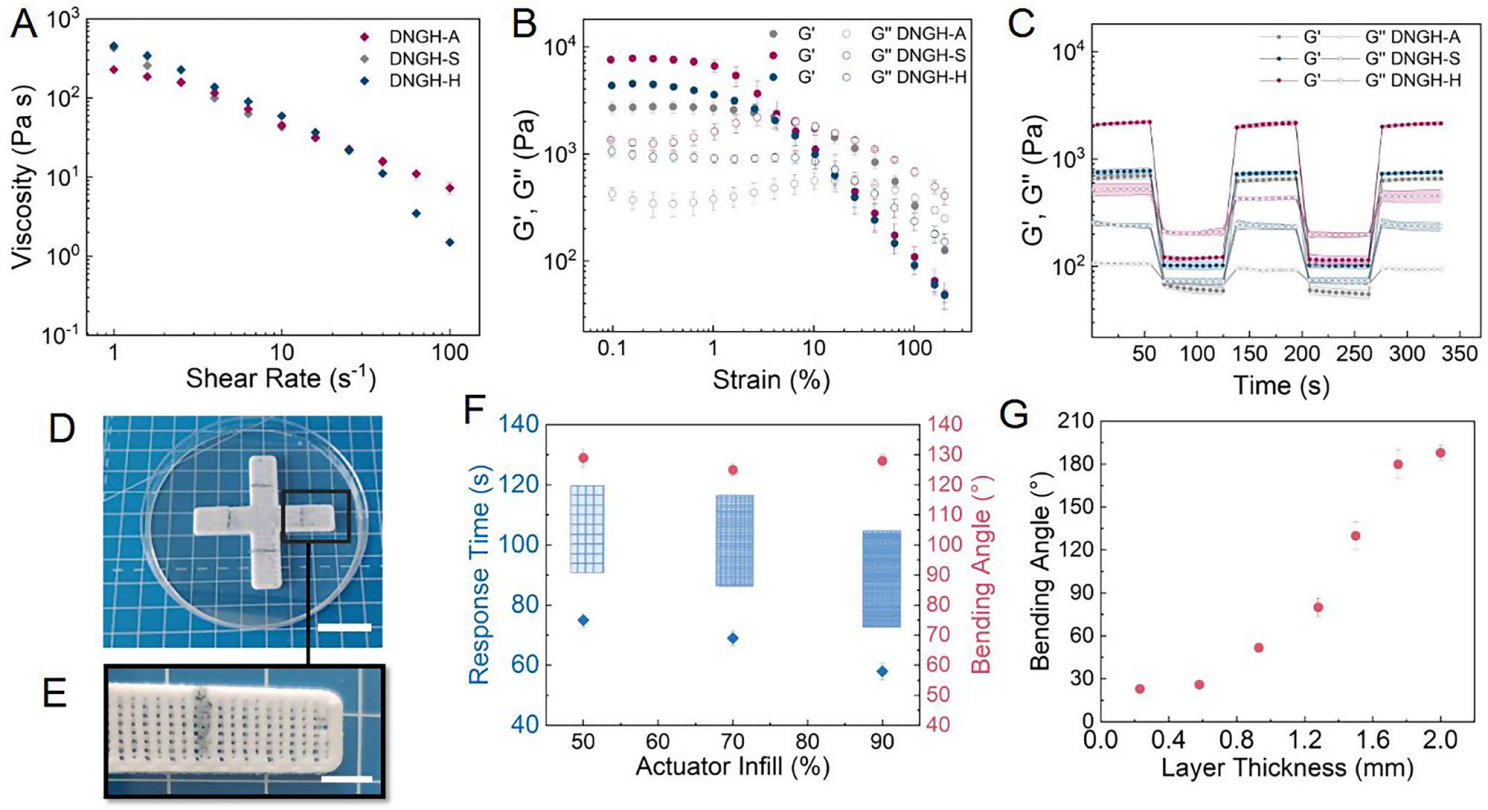
Rheological properties and co-3D Printing of DNGH actuator, sensor and heating element. Rheological characterization of inks used to fabricate the actuator (gray), sensor (blue), heating element (red). **A** Amplitude sweep, **B** shear rate and **C** shear recovery tests. **D** Photograph a DNGH gripper 3D printed with a 90% infill density and an integrated heating element (scale bar 1 cm). **E** Close up of a single leg of the 3D printed gripper (scale bar 5 mm). **F** Time required to reach the maximum bending (blue) and the maximum bending angle (pink) of actuators as a function of the infill density used to print them (**G**). Influence of layer thickness of actuators printed with a 90% infill density on the maximum bending angle.

3D printing enables cost-effective fabrication of customized structures and heating paths, which allow for more energy-efficient closed-loop actuation. To assess if our material can be 3D printed through direct ink writing, we characterize its rheological properties. All tested granular inks are shear thinning, as shown in Fig. 5A. They exhibit a low yield point, characterized as the crossover of the storage G’ and loss modulus G” in strain sweeps at 9% strain, as shown in Fig. 5B. In addition, they all display a fast shear stress recovery, as shown in Fig. 5C. These results indicate that the granular material fulfills the rheological requirements for direct ink writing. To leverage this feature, we direct ink write a dm scale gripper composed of a DNGH-A layer and a DNGH-S layer, as shown in Fig. 5D. The total fabrication time of this gripper is only 15 min. To enable closed-loop actuation, we 3D print the DNGH-H as a single line in the middle of the DNGH-A layer, as shown in Fig. 5E. Because both types of inks contain acrylamide-based monomers within their microparticles, we expect the free radical polymerization of acrylamides to proceed across the bilayer interface, yielding a strong interface.

To test if we can accelerate the actuation by introducing small open pores that facilitate the heat transfer into our grippers, we vary the geometrical infill density, while keeping the thickness of DNGH-Ss and DNGH-As constant at 1 mm. Indeed, the response time to achieve the maximum bending angle of 105 °C of our gripper increases from 160 to 190 s if we decrease the infill density from 90 to 50%, as shown in Fig. 5F, well in agreement with previous reports^39,40^. The smaller pores achieved with the higher infill enhance the heat transfer efficiency due to their higher surface area-to-volume ratio and improve the convective heat transfer, which accelerate response rates^41^. Importantly, changes in the infill density do not affect the maximum bending angle, as shown in Fig. 5F. Increasing the total length of the heating element leads to a decrease of the maximum temperature of the DNGH-H, compromising the activation of the PNIPAM, such that we do not investigate geometries with longer heating paths.

Grippers typically need to reach large bending angles. To assess if we can increase the maximum bending angle, we vary the thickness of our actuator printed with a 90% infill density while keeping the thickness of the DNGH-S constant at 1 mm. Indeed, the bending angle exponentially increases if the thickness of the actuator is increased beyond 0.6 mm, as shown in Fig. 5G. We associate this increase to a larger actuation force generated by thicker DNGH-A layers. A 1.8 mm thick actuator exhibits a maximum bending angle of 180°, which is desirable for our soft robotic gripper application. A further increase of the thickness to 2 mm does not yield a significant increase in the bending angle, most likely because the entire material becomes too stiff.

### Somatosensory gripper and closed-loop control

Somatosensory feedback of our soft robot gripper requires a reliable piezoresistive signal response. To verify the reliability of the DNGH-S sensor, we subject it to 20 cycles where the strain is varied between 0 and 30%. Within these cycles, we do not measure any signal drift, as shown in Fig. S11. Even if tested under quasi-static conditions by introducing a hold time of 30 s at maximum and minimum strain, the DNGH-S exhibits minimal signal drifts: In this case, the signal relaxes by 5% at maximum strain and not at all at minimum strain, as shown in Fig. S11. These results demonstrate the high reliability of the piezoresistive response of our sensor whose detection limit of the sensor is 0.5% strain, which is equivalent to 0.1° of bending.

**Fig. 6:**
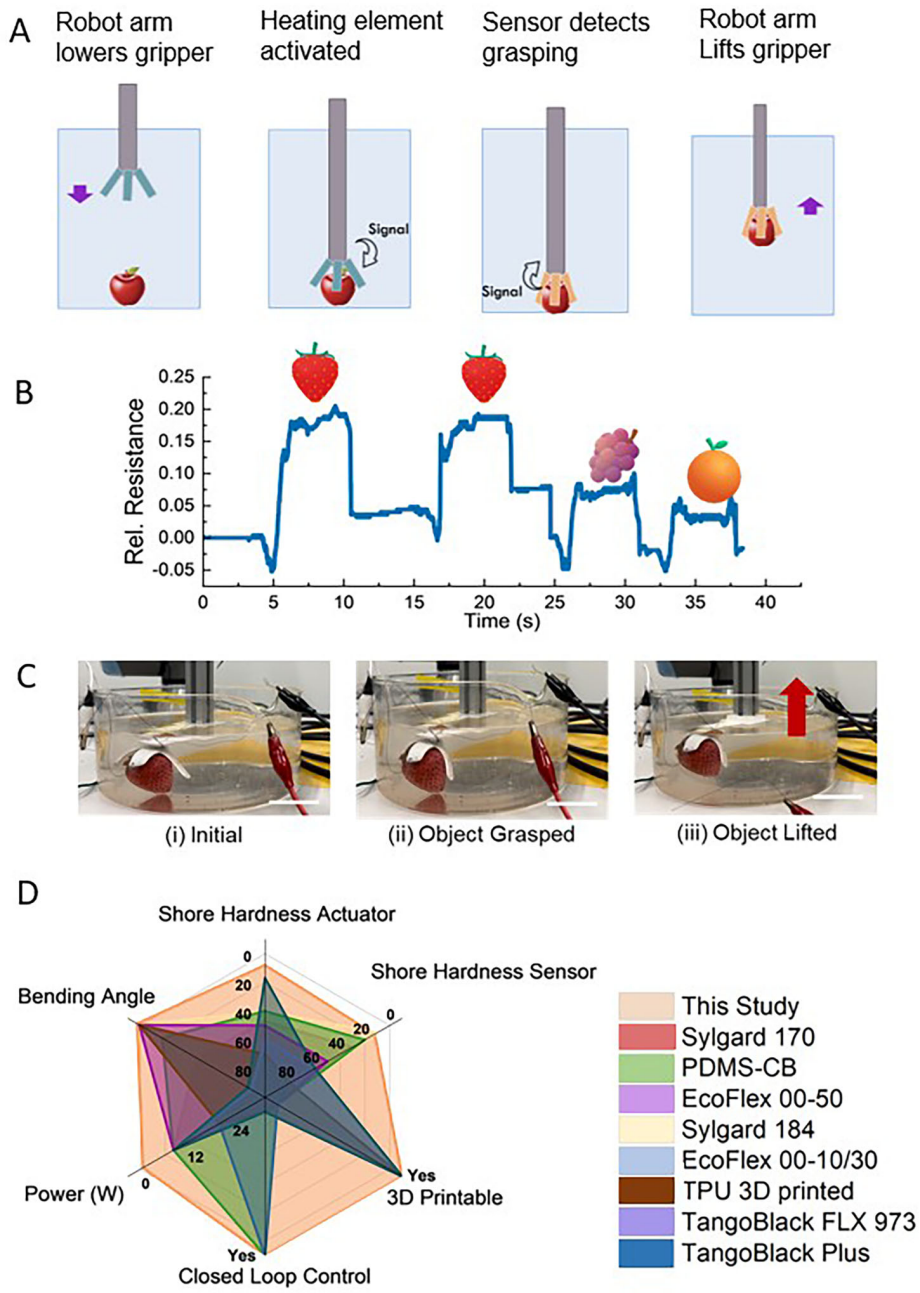
Somatosensory feedback and closed-loop control for DNGH gripper. **A** Schematic of the closed-loop control of the gripper relying on the somatosensory feedback. **B** Change in the relative resistance if the gripper picks up strawberries, grapes, and an orange. **C** Photographs of the gripper with the integrated somatosensory feedback (i) before (ii) after the heating is activated, such that a strawberry is gripped and (iii) lifted (scale bar 5 cm). **D** Radar chart of soft robotic grippers with integrated sensing depicting the soft actuator and soft sensor, bending angle, Shore hardness, their power consumption, 3D printability, and closed-loop control. The grippers consist of silicone elastomer polydimethylsiloxane and carbon black PDMS-CB (olive)^42^, silicone elastomer EcoFlex 00–50 (violet)^43^, silicone elastomer Sylgard 184 (yellow)^44^, silicone elastomer Sylgard 170 (magenta)^45^, thermoplastic polyurethane TPU 80 A (brown)^46^, silicone elastomer EcoFlex 00–10/30 (blue)^47^, rubber TangoBlack FLX 973 (teal)^48^, rubber TangoBlack Plus (purple)^49^, and the DNGH of this study (orange).

Soft grippers must be able to autonomously open and close. In addition, it is often advantageous if they can raise and lower their positions after gripping or releasing objects. To introduce this functionality into our gripper, we establish a closed-loop control between our gripper and a robot arm that relies on the proprioceptive feedback from the DNGH-S to recognize the time point when the desired object has been gripped, as schematically shown in Fig. 6A. We do not apply any voltage during the descent of the arm. When the robot arm is well positioned, voltage is supplied to the DNGH-H to activate the DNGH-A that then grips the object. The sensor signal from the DNGH-S records this motion, signaling the robot arm to lift the gripper with the object when the gripper reached its final gripping position. When the gripper reaches its release position, the controller withholds the voltage from the DNGH-H such that the DNGH-A slowly releases the object. During this action, the arm returns to its initial position where it releases the gripped object, as shown in Fig. S12.

Somatosensory feedback can be exploited to classify gripped objects. It should be noted that a 35 g DNGH gripper can lift up to three-fold its own weight, up to 110 g. To evaluate the possibility to recognize objects, we aim to classify strawberries among a selection of strawberries, oranges, and grapes. Contact with the object can be detected by a characteristic spike in the sensor signal at the onset of the hold phase, contributing to the soft robot’s somatosensory representation of object contact, as shown in Fig. 6B. When a 3 cm strawberry is gripped, the change in relative resistance, caused by an increased strain of the DNGH-S, is 0.18, as shown in Fig. 6B. Lifting larger fruits results in smaller changes in strain of the DNGH-S and hence, smaller variations in the relative resistance. Indeed, the relative resistance changes only by 0.08 if a 4.5 cm diameter cluster of grapes is gripped and by 0.05 if a 6 cm diameter orange is gripped. Based on those values, we program the controller to deactivate the heating element if the relative resistance is lower than 0.18, such that the gripper releases the object. By contrast, if the strawberry is gripped, the robot arm lifts, as shown in Fig. 6C and Movie 2. This approach enables reliable size- and shape-dependent handling for dissimilar fruits, such as strawberries and oranges. Such object recognition features enable sorting and packaging applications. To reliably separate more similar fruits, additional sensing and visualization entities would need to be added to the gripper.

Soft robotic grippers with closed-loop control and integrated somatosensory sensing have been previously reported. However, these grippers are traditionally based on tendon and pneumatic-based actuation, such that they cannot be 3D printed. Moreover, these grippers typically exhibit Shore hardnesses around 40 A. Our DNGH gripper for the first time combines closed-loop control, softness, and 3D printability, as summarized in Fig. 6D. The system operates using low-voltage electronics compatible with standard controllers and does not require pumps, compressors, or valves. Under our operating conditions, the embedded Joule heater consumes approximately 2 W during actuation. For context, pneumatic soft grippers typically rely on external pumps with rated power on the order of 10–12 W, assuming continuous operation. Direct comparisons across fundamentally different actuation mechanisms are constrained by differences in system architecture and reporting conventions. Nevertheless, this comparison highlights the advantage of thermoresponsive actuation if the heating element is incorporated in the thermoresponsive element.

## Discussion

We introduce soft grippers that can lift up to three times their own weight from double network granular hydrogels. These materials display Shore hardnesses below 10 A while reaching a bending angle of 180° within 90 s. Importantly, this bending is reached without significantly changing the surrounding temperature using a voltage as low as 5 V and a power as low as 2 W. We actuate the gripper by exploiting Joule heating, caused by a conductive double network granular hydrogel-based material (DNGH-H) that transforms electricity into localized heat. The localized heat causes PNIPAM to partially collapse, thereby inducing a bending of the gripper without excessively heating the surroundings. This feature clearly sets apart our DNGH-based actuator from more conventional PNIPAM-based counterparts that require heating of the bulk surrounding for actuation, rendering them less energy efficient and slower. Furthermore, integrated soft Joule heaters attribute electronic programmability to the gripper, a feature necessary for closed-loop control in soft robotics. The granular nature of the ink enables its direct ink writing into intricate multi-material 3D structures, such as a 15 cm sized gripper capable of lifting objects as heavy as 75 g if immersed in a water bath. Leveraging the piezoresistivity of the passive layer, we design a gripper that operates with a closed-loop control, which enables picking up a pre-defined element, such as a strawberry, from a fruit mixture. We foresee the combination of multi-functionality, programmability, softness, and processibility of our DNGH to open up new applications in energy-efficient autonomous soft robots.

## Methods

### Materials

N-isopropylacrylamide (NIPAM) (Carl Roth, Germany), Acrylamido-2-methylpropane sulfonic acid (AMPS) (Sigma-Aldrich, USA), an aqueous solution containing 30 wt% and 40% acrylamide (Fisher Scientific, USA), N,N-methylene bisacrylamide (MBA) (Carl Roth, Germany), 2-hydroxy-2-methylpropriophenone (Sigma-Aldrich, USA), zinc chloride (Sigma-Aldrich, USA), PEDOT:PSS (Sigma-Aldrich, USA) were used as received.

### Microgel and DNGH preparation

Bulk PAMPS samples were cast from an aqueous precursor solution containing 25 wt% AMPS, 5 wt% MBA relative to the total AMPS concentration, and 0.5 µl ml^−1^, 2-Hydroxy-2-methylpropiophenone that we use as a photoinitiator. To initiate the free radical polymerization, we expose the precursor to 1 mW cm^−2^ of 364 nm UV light for 5 min. PAMPS microgels were produced by fragmenting bulk hydrogels at room temperature using a grinder equipped with steel blades. To produce the double network granular hydrogel sensor (DNGH-S), PAMPS microgels were incubated overnight in an aqueous solution containing 30 wt% acrylamide, 2% MBA relative to the total polyacrylamide, 0.5 µl ml^−1^ PI, 0.027 wt% PEDOT:PSS. To produce the double network granular hydrogel Joule heating elements (DNGH-Hs), PAMPS microgels were incubated overnight in an aqueous solution containing 30 wt% acrylamide, 2% MBA relative to the total polyacrylamide, 0.5 µl ml^−1^ PI, 0.027 wt% PEDOT:PSS and 40 wt% ZnCl_2_.

Bulk PNIPAM samples were cast from an aqueous precursor solution containing 20 wt% NIPAM, 2 wt% MBA relative to the total NIPAM concentration, and 0.5 µl ml^−1^ 2-Hydroxy-2-methylpropiophenone PI. The cast samples were solidified by exposing them to 1 mW cm^−2^ of 364 nm UV light for 5 min to initiate the free radical polymerization of the precursors. PNIPAM microgels were produced by fragmentation at room temperature using a grinder equipped with steel blades. To produce the double network granular actuator (DNGH-A), PAMPS microgels were incubated overnight in an aqueous solution containing 20 wt% NIPAM, 2% MBA relative to the total NIPAM concentration, and 0.5 µl ml^−1^ PI. Microgels were jammed through centrifugation at 4500 *g* for 10 min. The supernatant was discarded. The resulting DNGH ink was cast or 3D printed before the samples were exposed to UV 364 nm, 1 mW cm^−1^ for 5 min to initiate the free radical polymerization of the precursors for the 2^nd^ network.

### Double network bulk preparation

Double network bulk samples for the sensor and heating element were prepared by casting an aqueous solution containing 25 wt% AMPS, 5 wt% MBA relative to the total AMPS concentration and 0.5 µl ml^−1^ PI. The films were polymerized by exposure to UV 366 nm, 1 mW cm^−1^ for 10 min. For the sensor, the films were swollen in an aqueous solution containing 25 wt% AAm monomer, 0.3% mol MBA, 0.5 µl ml^−1^ PI in relation to the AAm and 0.027 wt% PEDOT:PSS. For the heating element, samples were swollen in an aqueous solution containing AAm and 40 wt% ZnCl_2_. After overnight swelling of the microgels, the films were exposed to UV 366 nm, 1 mW cm^−1^ for 10 min to initiate the free radical polymerization.

Double network bulk actuators were prepared by casting an aqueous solution containing 20 wt% NIPAM, 2 wt% MBA, and 0.5 µl ml^−1^ PI. The films were polymerized by exposure to UV 366 nm, 1 mW cm^−1^ for 10 min. The films were incubated in an aqueous solution containing 20 wt% NIPAM, 2 wt% MBA, and 0.5 µl ml^−1^ PI. After overnight swelling, the films were exposed to UV 366 nm, 1 mW cm^−1^ for 10 min to initiate the free radical polymerization of the 2^nd^ network.

### Joule heating effect

To investigate the joule heating effect, a power source from PeakTech (PeakTech, Germany) was used to apply a voltage of 5 V. The temperature was measured with a DHT11(Adafruit Industries, New York, USA) and an Arduino microcontroller. The temperature of the surrounding water was measured with an infrared camera.

### Impedance spectroscopy

Two-probes electrical impedance spectroscopy was performed with circular samples of 1 cm diameter and 2 mm thickness, using a Gamry potentiostat (Gamry Instruments, USA). Two gold-plated glass slides were used as electrodes. The frequency of the alternating current (AC) was varied from 0.1 Hz to 0.1 MHz using 5 measurement points per decade. The amplitude of the signal is 10 mV. The equivalent circuit was fitted to the experimental data using the Gamry software (EChem Analyst 2, Gamry). The samples were tested at swelling equilibrium in deionized water. Three samples were measured per sample type, and plots represent the average of those three measurements.

### Rheological characterization

To characterize the rheology of the ink, shear rate sweeps, strain sweeps, and shear recovery tests were performed with a DHR-3TA Discovery instrument (TA Instruments, USA). An 8 mm parallel plate geometry was used with a gap of 0.8 mm. The TRIOS software from TA Instruments was used for recording the data. Amplitude sweep measurements were performed at 1.0 rad s^−1^ and subjected to strains over a range of 0.01–300%. Shear thinning measurements were performed in the rotational mode with a strain rate between 1 and 100 s^−1^. Shear recovery measurements were performed at 1.0 rad s^−1^ and strains of 1 and 30% were applied alternatively for 60 s. The cycle was repeated two times.

### Tensile testing and piezoresistive sensor response characterization

To fabricate samples for tensile testing, the DNGH-S hydrogel was cast into dog-bone-shaped Teflon molds with a cross-section of 4.6 mm^2 ^^2^. Tensile tests were performed with a Zwick Roell Z005 universal testing machine (Zwick Roell, Ulm, Germany) with a strain rate of 200 mm/min. To characterize the piezoresistive response, ten cycles of 0–30% strain and 0–100% strain were performed while simultaneously recording the electrical resistance with an Arduino microcontroller equipped with an analog-to-digital converter (ADC) unit. Quasi-static measurements were performed by applying five cycles of 0–30% strain with a dwell time of 30 s applied at maximum and minimum strains. The drift was calculated as the percentage difference of the relative resistance value at maximum strain between the second and last cycle. The relaxation was calculated as the percentage difference of the electrical resistance at the beginning and end of the dwell time, during the quasi-static measurements.

### 3D printing

A BIO X bioprinter (CellInk, USA) equipped with three extruders was used to fabricate actuator strips and the soft robotic gripper in a one-step process. The ink was extruded with a 20 G conical nozzle (0.603 mm diameter) through a pressure-driven piston with a speed of 10 mm s^−1^.

### UV-VIS spectroscopy

UV–Vis spectroscopy of the supernatant of jammed microgels was performed between 200 and 700 nm with a Perkin Elmer Lambda 365 spectrophotometer (Perkin Elmer, USA).

### Tests with gripper and robot arm

A Universal Robots UR5 robotic arm was used for lowering and lifting the gripper with a speed of 10 mm/s. The closed-loop control was established linking the sensor response of the gripper with the lifting and lowering function of the robot arm at a control frequency 200 Hz.

## Supplementary information

Supplementary_information_R1_V2_no_markups

gripper_montage_strawberry_orange

soft-medium_hard_strips

## Data Availability

Data for this article, including electromechanical characterization data, rheological characterization data, impedance spectra, bending angles during actuation and sensor signals are available at Zenodo: 10.5281/zenodo.15922612.
