# Supplementary material for: Programmable somatosensory soft robots
Source: Npj Flex Electron. 2026 Mar 7;10(1):58. doi: 10.1038/s41528-026-00558-0 (PMC13095659; doi:10.1038/s41528-026-00558-0)
Supplement: Supplementary file 1 — Supplementary_information_R1_V2_no_markups [file 41528_2026_558_MOESM1_ESM.pdf]

Supplementary Information

Programmable and somatosensory thermoresponsive soft robots

Antonia Georgopoulou[1,2], Malena Aguiriano Calvo[1,3], Lorenzo Lucherini[1], Sudong Lee[3], Josie Hughes[3], Esther Amstad[1,2]

1) Soft Materials Laboratory, Institute of Materials (SMaL), École Polytechnique Fédérale de Lausanne, 1015 Lausanne, Switzerland.

2) Swiss National Center for Competence in Research (NCCR) Bio-inspired Materials, University of Fribourg, Chemin des Verdiers 4, 1700 Fribourg, Switzerland.

3) CREATE Lab, Institute of Mechanical Engineering, École Polytechnique Fédérale de Lausanne, 1015 Lausanne, Switzerland.

E-mail: esther.amstad@epfl.ch

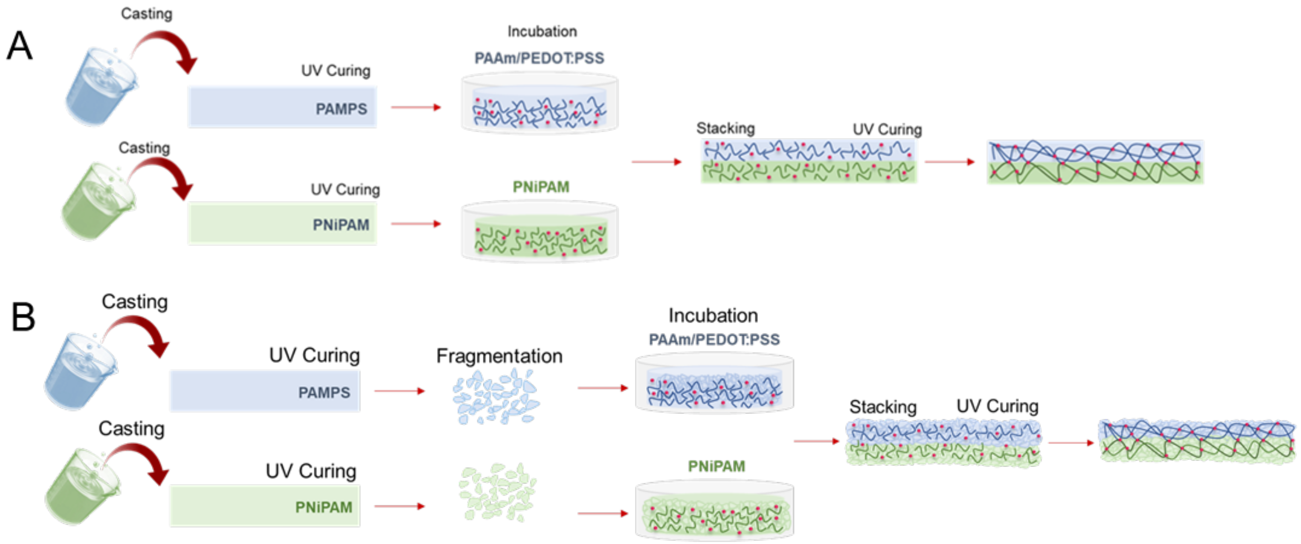

Fig. S1. Schematic illustration of the preparation of the actuators based on **A.** double network bulk and **B**. DNGH hydrogels.

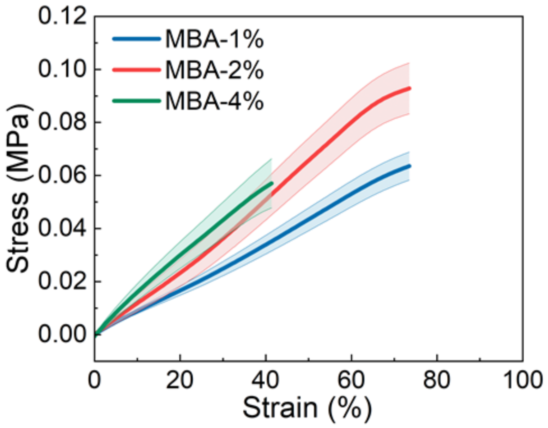

Fig. S2. Stress-strain curves of the DNGH-A composed of PNIPAM fragments containing 1 wt% (blue), 2 wt% (red) and 4 wt% (green) MBA crosslinker.

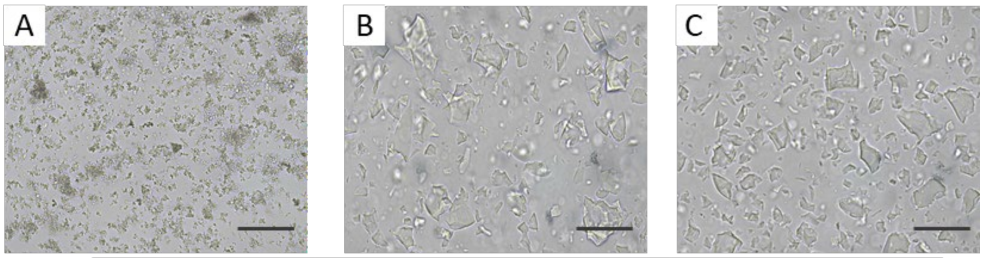

D

	Average Diagonal Length (mm)
DNGH-A	11 ± 4 μm
DNGH-S	65 ± 13 μm
DNGH-H	59 ± 14 μm

Fig. S3. Optical micrographs of **A**. PNIPAM microgels incubated in a NIPAM containing aqueous solution **B**. PAMPS microgels incubated in a AAm/PEDOT:PSS containing aqueous solution C. PAMPS microgels incubated in an aqueous solution containing AAm, PEDOT:PSS and $ZnCl_2$ (Scale bars 100 μm). **D**. The average diagonal length derived from A,B,C.

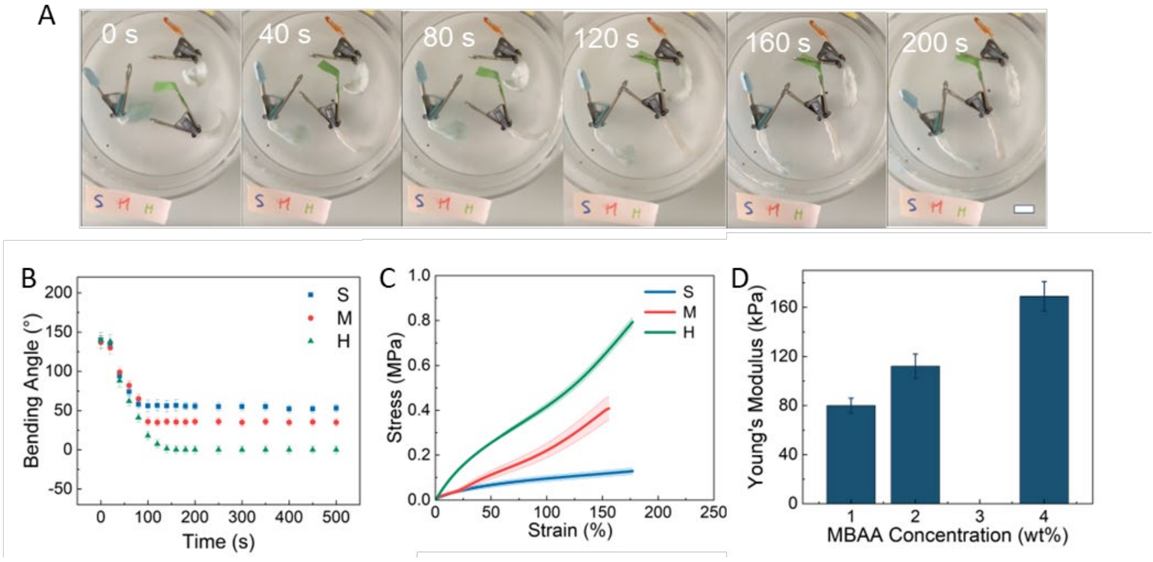

Fig. S4. A. Timelapse of composite strips immersed in 55°C water. The DNGH-S contains 2 wt% (S), 3.5 wt% (M) and 5 wt% (H) MBA crosslinker (scale bar 5 mm). **B.** Evolution of bending angle with time of composite strips immersed in 55°C water. The DNGH-S contain 2 wt% (blue), 3.5 wt% (red) and 5 wt% (green) MBA crosslinker. **C.** Stress-strain curves of DNGH-S containing 2 wt% (blue), 3.5 wt% (red) and 5 wt% (green) MBA crosslinker. **D**. The Young's modulus as a function of the MBA crosslinker concentration in the microfragments.

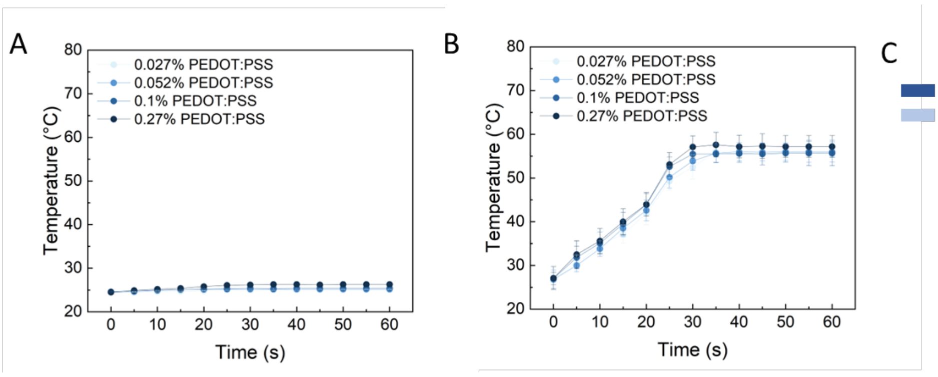

Fig. S5. Temperature evolution of single network bulk PAAm gels as a function of the PEDOT: PSS concentration within them if subjected to 5V **A**. without and **B**. with 40 wt% $ZnCl_2$. **C**. Schematic of the positioning of the Joule heater on top of the actuator.

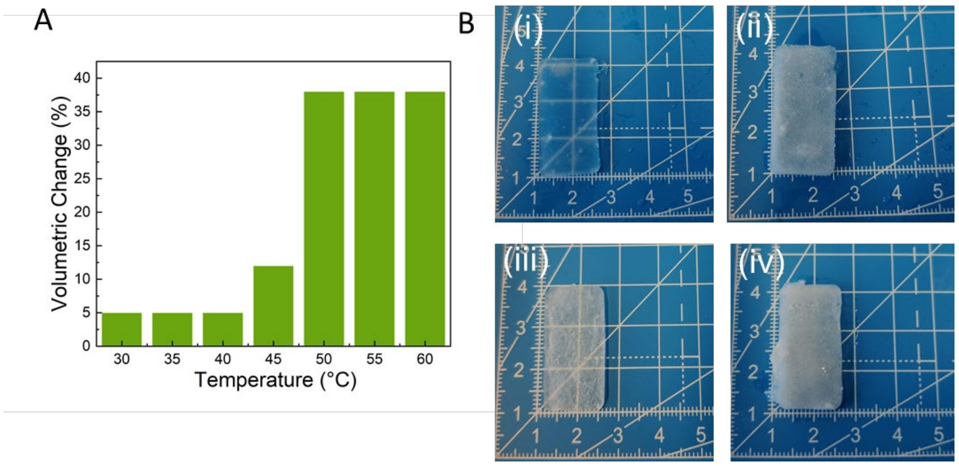

Fig. S6. A. Volumetric change of PNIPAM strips containing 1 wt% MBA crosslinker as a function of temperature when before and after immersion to 55°C water. **B.** Photographs of PNIPAM strips with (i, ii) 1 wt% and (iii, iv) 4 wt% MBA crosslinker at (i, iii) 25°C and (ii, iv) 50°C.

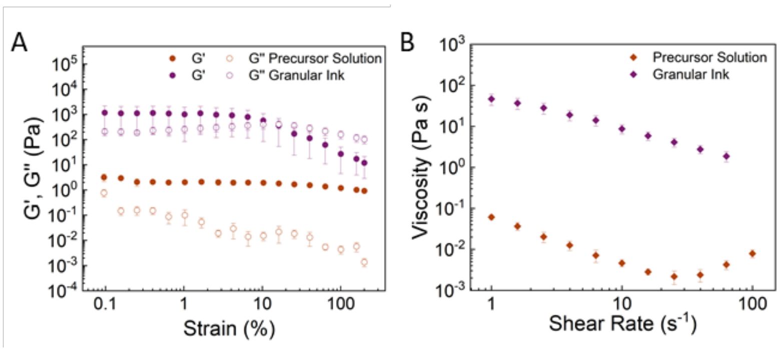

Fig. S7. Rheological properties of the PAAm precursor solution (orange), PAMPS/PAAm granular ink (purple) **A.** amplitude sweep and **B.** shear rate.

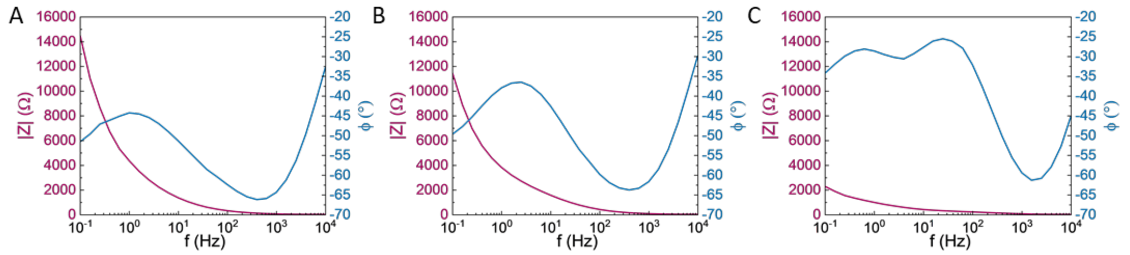

Fig. S8. Bode plots featuring the impedance (red) and phase (blue) obtained during impedance spectroscopy of **A.** PAMPS-PAAm DNGHs **B.** PAMPS-PAAm DNGHs functionalized with PEDOT:PSS (DNGH-S) **C.** PAMPS-PAAm DNGHs functionalized with PEDOT:PSS and Zn^{2+} (DNGH-H).

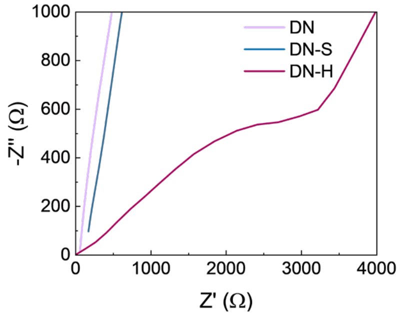

Fig. S9. Nyquist plots obtained with impedance spectroscopy for double network bulk (DN), bulk DN-H containing 40 wt% $ZnCl_2$ and 0.027 wt% PEDOT:PSS and DN-S containing 0.027 wt% PEDOT:PSS.

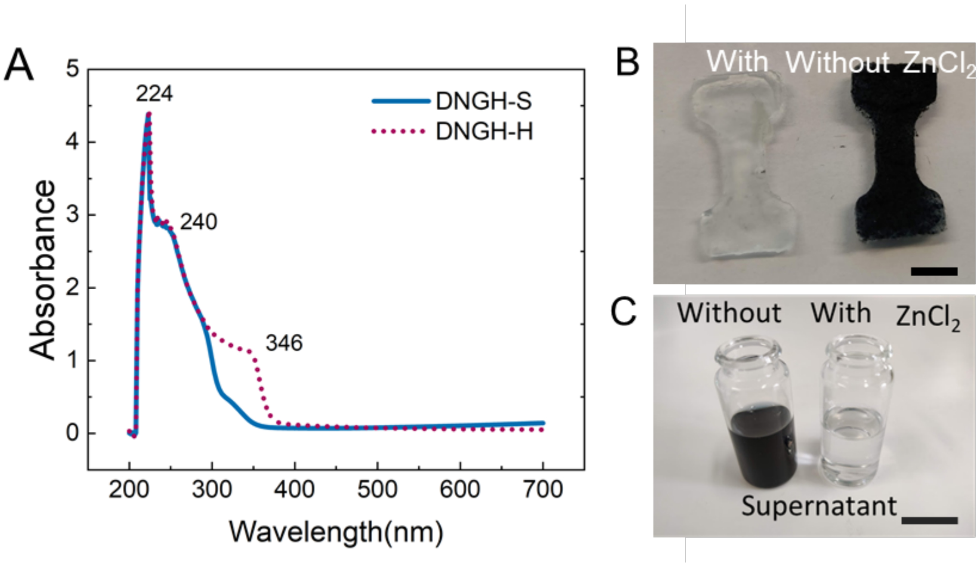

Fig. S10. A. UV-VIS Spectra of the supernatant of jammed microgels used to fabricate DNGH-S (blue) and DNGH-A (red). **B.** Photographs of DNGH-S (left) and DNGH-H (right)-scale bar 1 cm **C.** Photograhs of the supernatant jammed microgels used to make DNGH-S (left) and DNGH-H (right)-scale bar 1 cm.

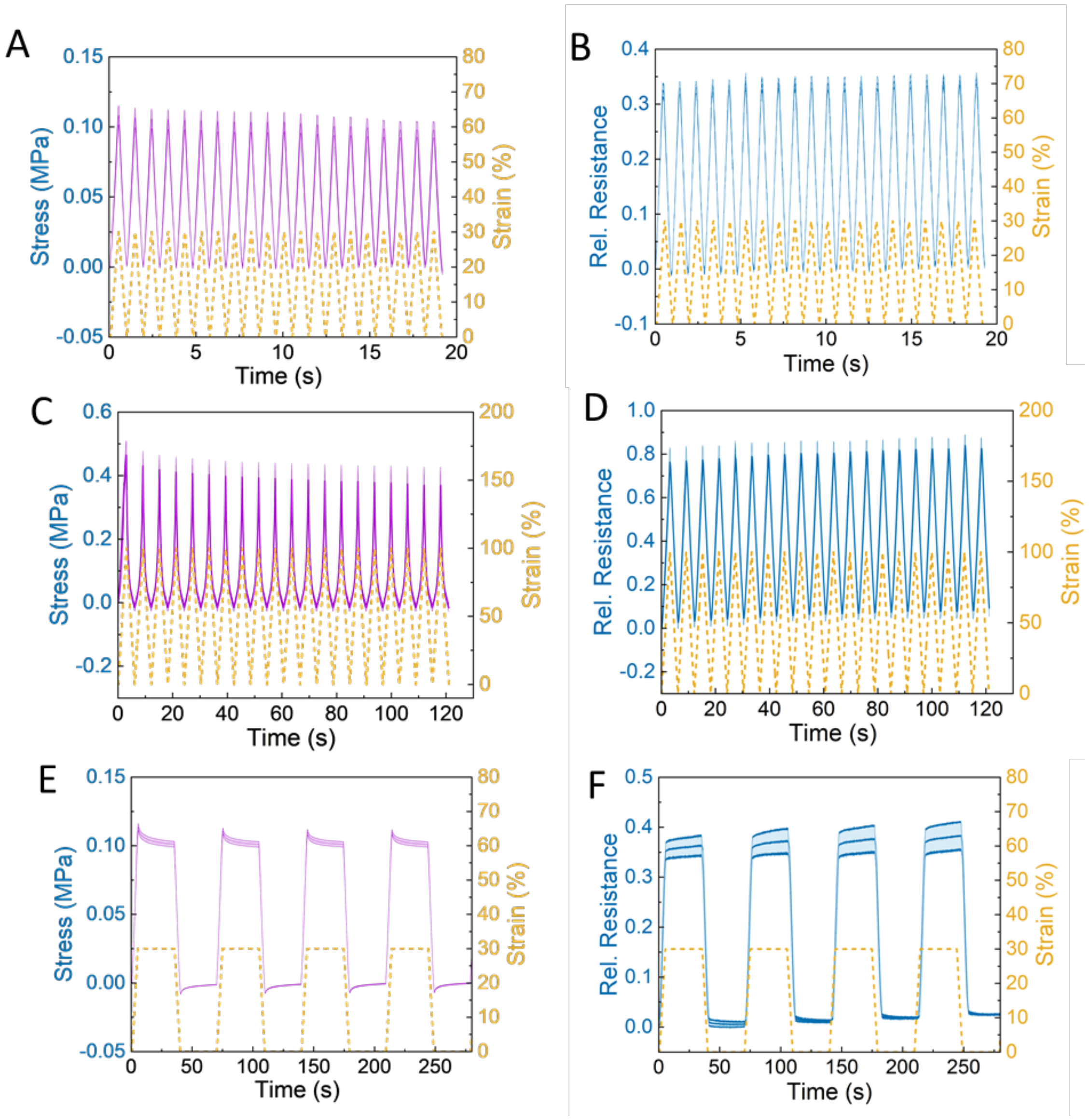

Fig. S11. Piezoresistive sensor signal (blue), mechanical stress (purple) and strain (yellow) during the mechanoelectrical characterization of the DNGH-S with 5 wt% MBA, involving: **A**. Stress response and **B**. Relative resistance response during 10 cycles between 0-30% strain. **C**. Stress response and **D**. Relative resistance response during 10 cycles between 0-100% strain. **E**. Stress response and **F**. Relative resistance response during 5 cycles between 0-30% strain at maximum and minimum strain.

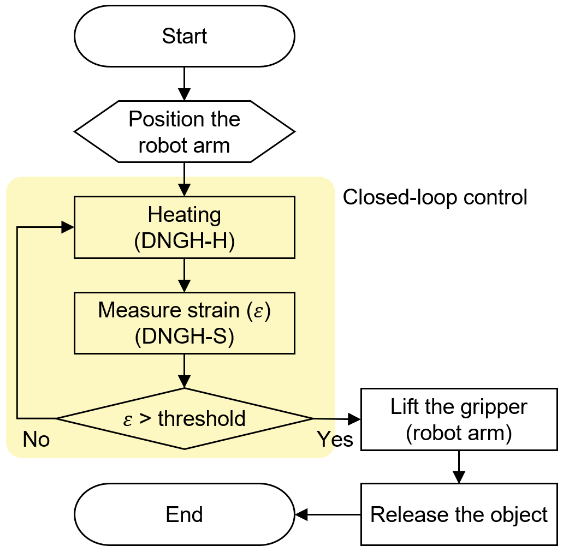

Fig. S12. Closed-loop control linking the heating, sensor response and positioning of the robot arm.

Movie 1. DNGH bilayers at 55°C with DNGH-S containing contain 2 wt% (S), 3.5 wt% (M) and 5 wt% (H) MBA crosslinker.

Movie 2. DNGH gripper with closed-loop control picking up an orange and a strawberry. The gripper is programmed to pick up the smaller strawberry thanks to its somatosensory feedback.
